# A rare case of adrenal extramedullary haematopoiesis in a Cypriot woman with β-thalassaemia

**DOI:** 10.1308/rcsann.2021.0298

**Published:** 2022-01-04

**Authors:** AC Georgiou, AB Lisacek-Kiosoglous, D Mariannis, S Christou, VG Hadjianastassiou

**Affiliations:** ^1^ European University Cyprus, Nicosia, Cyprus; ^2^ Makarios Thalassemia Hospital Nicosia, Cyprus; ^3^ Makario Children’s Hospital Nicosia, University of Nicosia Medical School, Nicosia, Cyprus; ^4^ Bart’s Health NHS Trust, UK

**Keywords:** General surgery, Adrenal Surgery - Thalassaemia

## Abstract

We report a rare case of adrenal extramedullary haematopoiesis (EMH) in a thalassaemia patient in Cyprus. A 40-year-old woman with β-thalassaemia presented with a 2-day history of non-specific right-sided abdominal pain on routine follow-up for her thalassaemia treatment. Her laboratory tests were not dissimilar to her routine results and no palpable mass was detected. Computed tomography findings revealed a 5.8×4.2×4.6cm solid lesion in the right adrenal gland. Surgical excision was advised for this symptomatic large tumour with the possibility of malignancy in a young patient, and a laparoscopic adrenalectomy was performed. Postoperative follow-up was uneventful. A review of the literature in PubMed and MEDLINE revealed 14 case reports worldwide with adrenal EMH secondary to β-thalassaemia. EMH tumours in patients with thalassaemia have been reported incidentally, which stresses the importance of considering this in the list of differentials of adrenal incidentalomas in this patient population.

## Background

Beta-thalassaemia is a genetic condition of the HBB gene on chromosome 11 which results in abnormal β-globin chains.^1^ It is inherited in an autosomal dominant fashion that can result in a range of outcomes from severe anaemia and iron overload to clinically asymptomatic individuals.^1^ Three main forms of β-thalassaemia exist, major, media and minor.

Haematopoietic stem cells in the bone marrow are responsible for the production of red blood cells. When haematopoiesis occurs outside the bone marrow it is referred to as extramedullary haematopoiesis (EMH).^2^ EMH occurs secondarily to altered bone marrow function and subsequent abnormal haematopoiesis.^2,3^ As reported by Keikhaei *et al*,^4^ EMH is caused by a long-standing response due to severe chronic anaemia. Maryam *et al*^2^ reported haemolytic anaemia and myelofibrosis as a cause of EMH. The most common sites of EMH are reported^2,3^ to be the spleen, liver, lymph nodes, lung, pleura, breast, thymus, small bowel and central nervous system. EMH is rarely found in the kidneys and adrenal glands.^2,3^

Haemoglobinopathies, haemolytic anaemias (such as thalassaemia), leukaemias, lymphomas and myeloproliferative disorders are examples of conditions that have reported complications of adrenal EMH requiring surgery.^2^ Imaging and adrenal hormonal investigations are important to exclude malignancy and subclinical hypersecretory syndromes.^3^

The aim of this case report is to further document and call attention to the potential diagnosis of adrenal EMH tumours in patients with β-thalassaemia. As shown in our literature review, and including the patient described here, only 14 cases of adrenal EMH tumours have been reported worldwide in the literature in patients suffering from β-thalassaemia. Notably, seven cases were found incidentally, indicating the potential for adrenal tumours in patients with β-thalassaemia. Without biopsy and monitoring, these tumours have the potential to go undiagnosed until a later date, which may be detrimental to the patient’s prognosis. This report highlights the importance of being cognisant of EMH tumours in the adrenal gland (although rare) to ensure optimal prognostication of the patient. Here, a rare case of EMH in Cyprus is described, resulting in laparoscopic adrenal surgery due to diagnostic uncertainty and the patient’s symptoms.

## Case history

A 40-year-old woman from Cyprus presented for routine follow-up at Makarios Thalassemia Hospital, where she had been receiving regular treatment for β-thalassaemia. The patient reported a 2-day history of non-specific right-sided abdominal pain.

**Figure 1:**
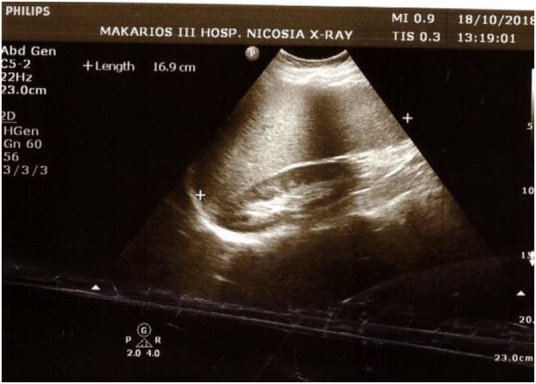
Ultrasound image of the liver and right kidney

**Figure 2:**
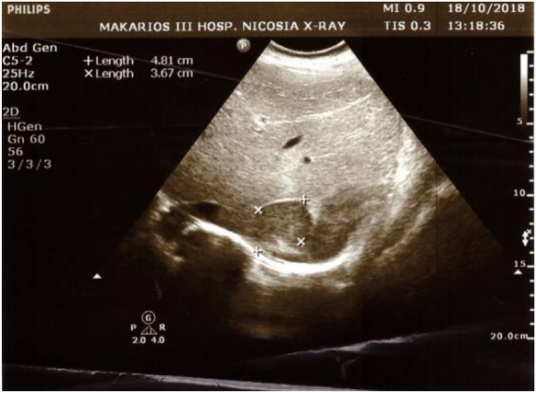
Ultrasound image of the liver and adrenal mass x/+ indicating dimensions

**Figure 3:**
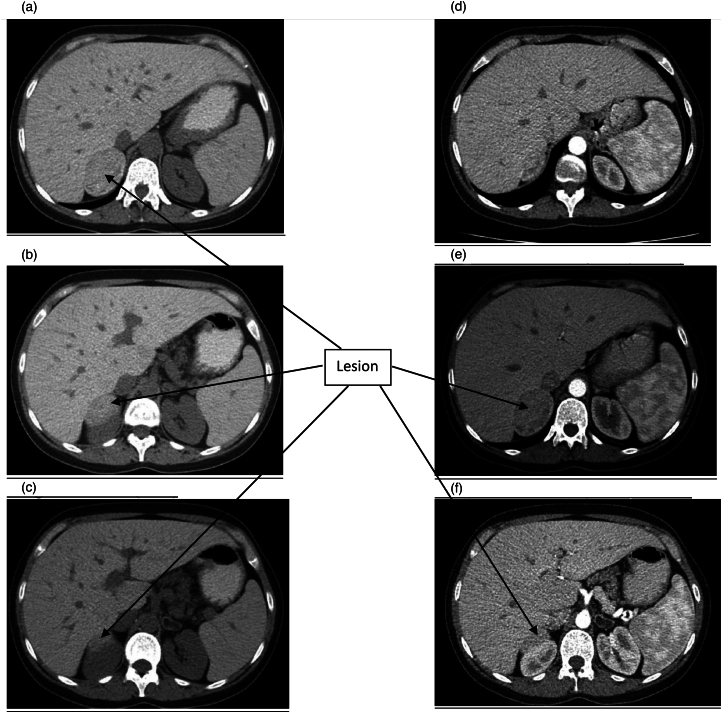
Computed tomography scans: (a) no contrast (NC) (H=−65), (b) NC (H=−80), (c) NC (H=−85), (d) arterial phase H=−58.5, (e) arterial phase H=−73.5, (f) H=−92.9. H = Hounsfield units.

She returned to the hospital and on physical examination reported tenderness in the right flank with no palpable mass. No tenderness had been reported on previous physical abdominal examinations. On palpation, splenomegaly was found as per previous follow-ups over the years. The right-sided flank pain led to further investigation with ultrasound and computed tomography (CT), and an adrenal mass was detected (Figures 1–3). Findings included hepatosplenomegaly (liver, 21cm; spleen, 19.5cm).

The patient denied any symptoms such as headaches, changes in skin, palpitations, hypertension, weakness, weight loss or urinary tract symptoms.

The patient’s dietary habits had not changed and included avoidance of meat while still achieving a balanced diet overall. Her mobility prior to surgery was reported as normal.

### Timeline of past medical history

The patient had a long-standing diagnosis of β-thalassaemia Homozygote IVSI-110 since 2002 and was receiving regular outpatient treatment. She routinely received 2 units of packed red blood cells, twice per month (4 units per month) for β-thalassaemia and had regular blood analyses every 3 months. On routine examination she was found to have non-tender splenomegaly (∼14cm detected with ultrasound) as per patients with thalassaemia. Her relevant routine laboratory findings since 2016 are given in Table 1.

**Table 1:** Key laboratory findings for the period 2016–2018

	**Year**								
	**2016**		**2017**			**2018**			
Blood transfusions (*n*)	32		31			28			
Packs (units)	58		61			55			
Mean blood transfusion (ml)	310.4		292.5			290.8			
Volume (ml)	12,151		12,636			11,890			
Volume (ml/kg)	202.52		200.57			182.92			
Weight (kg)	60		63			65			
Date	30 November 2016		20 April 2017	23 June 2017	14 August 2017	8 March 2018	16 August 2018	31 December 2018*	Normal values
Lactate dehydrogenase (units/l)	386		398	460	364	312	297	316	208–378
Total bilirubin (mg/dl)	3		4.09	2.98	4.13	4.81	3.74	3.17	0.20–1.00
Direct bilirubin (mg/dl)	0.52		0.57	0.5	0.69	0.7	0.6	0.5	0.00–0.20
WBC (1,000/μl)	5.11	4.53	4.99	5.58	7.57	6.37	5.19	7.52	3.91–8.77
Haemoglobin (g/dl)	9.2	8.7	9.3	8.7	10.3	9.9	9.7	10.4	11.5–15.5
Neutrophils (1,000/μl)	2.92	2.54	3.04	3.2	4.59	3.94	3.13	4.8	1.82–7.42
Platelets (1,000/μl)	141	198	158	148	192	131	154	163	150–450
Ferritin (ng/μl)	–	–	6,932	6,406	7,955	8,120	7,484	–	20–320ng/ml (Male) 12–150ng/ml (female)

*Postoperative results

Routine T2-weighted magnetic resonance imaging studies of the heart and liver in 2011–2018 revealed good physiological function and normal levels of iron in the heart, but the liver was seen to have a steady increase in haemosiderin. The adrenal lesion had not been identified in those scans. Overall, her levels of haemosiderin were above the normal range.

In October 2018, the patient presented with right-sided flank pain and subsequent examination led to ultrasound (Figures 1 and 2). CT findings included a solid lesion measuring 5.8×4.2×4.6cm (Figure 3), with mild heterogeneity, in the upper region of the right adrenal gland. There were no signs of extra fluid accumulation or lymph node involvement. After confirming a normal endocrine adrenal axis profile, a surgical consult was recommended. In view of the large size of the tumour and heterogeneity in the cross-sectional imaging, and the patient’s young age, the potential for malignancy could not be excluded. Surgical excision was advised without prior percutaneous biopsy as per the recommendations of the European Society of Endocrinology.^5^ The patient was admitted to hospital on the day of the operation. Surgery with a view to establish a tissue diagnosis was performed in late November 2018.

### Laboratory findings

As seen in Table 1, blood analysis showed increased bilirubin and decreased haemoglobin (Hb) secondary to β-thalassaemia. These are expected values for a patient with β-thalassaemia. The lactate dehydrogenase values decreased to within normal range from late 2017. White blood cells and neutrophils were in normal range and thus did not indicate any abnormalities outside the normal range for β-thalassaemic patients. Ferritin levels were very high. Endocrine adrenal axis tests included 24-hour urine tests, such as vanillylmandelic acid, metanephrine and normetanephrines, which were all within the normal range prior to surgery.

### Surgery

After securing informed consent, the patient underwent a right laparoscopic adrenalectomy. The surgical procedure included a laparoscopic adhesiolysis – due to previous (cholecystectomy) adhesions of small bowel and omentum on the liver. Laparoscopic mobilisation of the liver and right colon flexure was then done medially to reveal the right kidney. Following that, dissection of the inferior vena cava and right renal vein and laparoscopic right adrenalectomy (en bloc with the mass arising from it) was performed using a Thunderbeat scalpel. The procedure went smoothly without any complications and the patient was discharged home after two nights in hospital. The patient’s original pain resolved and follow-up at two years did not suggest a recurrence. Clinically she had fully recovered.

### Histopathology

**Figure 4:**
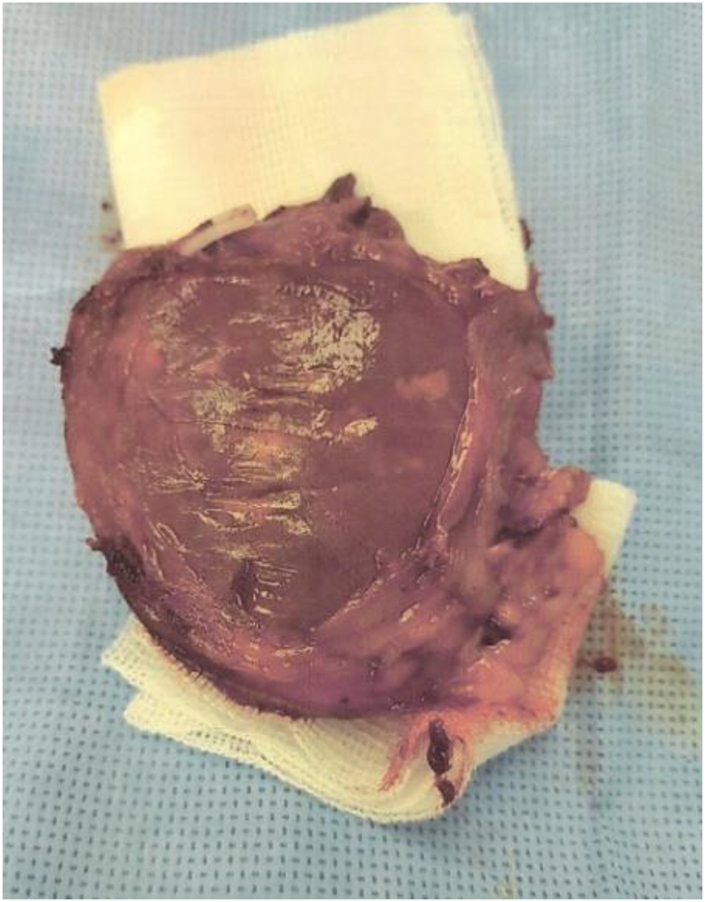
Histological specimen: right adrenal mass

**Figure 5:**
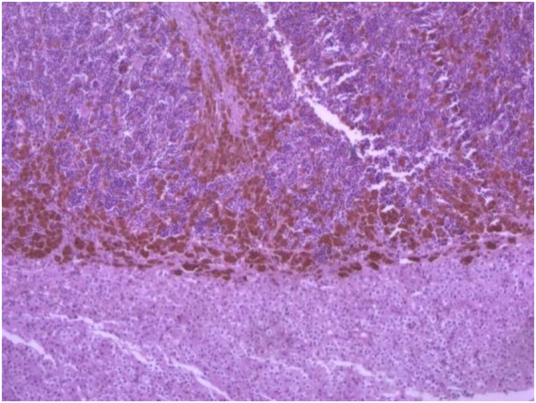
Histological imaging of the adrenal mass.

A 5.5cm adrenal mass was found, as seen in Figure 4. The specimen was received in formalin and labelled with the patient’s name and ‘RT adrenalectomy’. The specimen weighed 30g and measured 7.5×5×2.5cm surrounded by yellow/white unremarkable adipose tissue. There was a centrally located mass measuring 4cm. The lesion was brownish in colour, haemorrhagic and fragile. Histopathology examination revealed features of EMH associated with the patient’s known thalassaemia as seen in Figure 5.

The lesion was characterised by hematopoietic cells (myeloid, erythroid, megakaryocytic, lymphoid) and occasional adipocytes. The differential diagnosis included adrenal myelolipoma. However, myelolipoma is characterised by larger amount of adipose tissue and is not associated with haematological disorders. No evidence of malignancy was detected.

## Discussion

A literature review was conducted in PubMed and MEDLINE, using key words ‘Extramedullary Haematopoeisis’ and ‘Thalassaemia’. After searching the literature for similar studies it appears that only 14 other case reports (15 including this case) of adrenal EMH tumours have been reported from various countries where β-thalassaemia is prevalent, including Iran, China, Greece, Taiwan, Italy, Qatar and India (Table 2). Furthermore, an additional six case reports of adrenal EMH tumours associated with other haemoglobinopathies have been reported in China, Greece, Iran and the USA (Table 3).

**Table 2:** Summary of reported thalassaemia cases in literature review (databases: PubMed and MEDLINE)

Study (year)	Patient origin	Age (years)	Gender	Location	Thalassaemia type	Haemoglobin (g/dl)	Symptoms	Size (cm)	Management	Follow-up
Papavasiliou *et al* (1990)^6^	Greece	16	Male	Right	Thalassaemia	8.2	Incidentaloma	–	Surgical adrenalectomy	1–6 years
Wat *et al* (1998)^7^	China	31	Male	Bilateral	Beta-thalassaemia Intermedia	8	Abdominal pain	–	–	–
Chuang *et al* (1998)^8^	Taiwan	27	Female	Right	Beta-thalassaemia	–	Palpable non-tender abdominal mass	7.5×5.8	Surgical exploration, biopsy and conservatively	–
Porcaro *et al* (2001)^9^	Italy	10	Female	Right	Beta-thalassaemia	10	Incidentaloma	5	Surgical open adrenalectomy	72 months. Patient died because of disease complications related to infection and heart failure
Keikhaei *et al* (2012)^4^	Iran	26	Male	Right	Beta-thalassaemia Major	7.2	Incidentaloma	7.7×7.3×6.8	Medical treatment with hydroxyurea and blood transfusion	2 months
Maryam *et al* (2013)^2^	Iran	23	Male	Right	Beta-thalassaemia Major	–	Incidentaloma	13×11×5	Surgical open adrenalectomy	6 and 12 months
Banergi *et al* (2013)^10^	India	40	Male	Right	Homozygous delta–beta-thalassaemia	11.2	Upper abdominal pain and anorexia	8.9	Laparoscopic adrenalectomy	–
Karami *et al* (2014)^11^	Iran	23	Male	Right	Beta-thalassaemia	–	Incidental mass on ultrasound and CT	10×12	Surgical open adrenalectomy	–
Sekar *et al* (2015)^12^	India	29	Male	Right	Beta-thalassaemia	6.7	Anaemia, Jaundice and incidentaloma	8.4×7	Conservatively, 3U PRBs, oral hydroxyurea 500mg and folic acid, improving Hb to 9.3g/dL	6 month follow up. Still asymptomatic.
Zeighami *et al* (2015)^13^	Iran	33	Male	Left	Beta-thalassaemia Major	11	Weight loss, loss of appetite a few months prior.	9×7×7	Surgical open adrenalectomy	–
Al-Thani *et al* (2016)^3^	Qatar (Pakistani heritage)	48	Female	Right	Beta-thalassaemia Trait	10.8	Abdominal pain, fullness and palpable abdominal mass	16.6×11.7×10.4	Adrenalectomy	2 months
Motta *et al* (2016)^14^	Italy	40	?	Right	Beta-thalassaemia Major, genotype b0 cod39/b1 IVSI-110	–	Big palpable mass	19×15×25	Surgery	–
Kannan *et al* (2017)^15^	India	21	Female	Right	Heterozygous Hb E-thalassaemia	5.3	Right upper quadrant abdominal pain	8×7	Adrenalectomy	–
Tanner *et al* (2017)^16^	–	30	Male	Right	Beta-thalassaemia	10.5	Epigastric pain	4	Laparoscopic cholecystectomy and adrenalectomy	–
Georgiou *et al* (2018) – This study	Cyprus	40	Female	Right	Beta-thalassaemia Major–Homozygote IVSI-110	10.4	Right upper quadrant pain, no palpable mass	7.5×5×2.5	Adrenalectomy	1 month

**Table 3:** Summary of reported cases (other haemoglobinopathies) in literature review (Databases: PubMed and MEDLINE)

Study (year)	Patient origin	Age (years)	Gender	Location	Haematological disorder	Haemoglobin (g/dl)	Symptoms	Size (cm)	Management	Follow-up
King *et al* (1987)^17^	USA	66	Female	Bilateral	Agnogenic myeloid metaplasia	–	Incidentaloma following surgery	2.5×4.0	Needle biopsy	5 weeks: died from perforated gastric ulcer from uncontrolled haemorrhage
Calhoun *et al* (2001)^18^	USA	9	Male	Right	Hereditary spherocytosis	–	Jaundice Incidentaloma	5.5×5×2	Partial adrenalectomy	–
Arkadopoulos *et al* (2009)^19^	Greece	75	Female	Left	–	–	Breast cancer and adrenal incidentaloma (adrenal cavernous haemangioma with extramedullary haemopoietic tissue	8×6×4	Surgical open adrenalectomy	–
Lau *et al* (2011)^20^	China	43	Female	Right	Hb H constant Spring disease	–	Anaemia, jaundice, and right upper quadrant pain	7.5	Surgical open adrenalectomy	–
Zhao *et al* (2012)^21^	China	22	Female	Right	Hereditary spherocytosis	8	Weakness and jaundice for 3 months, anaemia during pregnancy	6.6	Surgically removed post delivery	–
Azarpira *et al* (2014)^22^	Iran	15	Female	Left	Homozygous sickle cell	11	Multiple painful bone crises and one acute splenic sequestration	7×5×3	Surgical open adrenalectomy	–

The haemoglobinopathies associated with adrenal EMH are β-thalassaemia,^3,10,12–16^ HbH constant spring disease (thalassaemia-α),^20^ HbE homozygous thalassaemia,^15^ hereditary spherocytosis^18,21^ and sickle cell.^6,22^ Thalassaemia was the most consistent disorder associated with adrenal EMH. King *et al*^17^ cited a different association, one with agnogenic myeloid metaplasia, a rare myeloproliferative disorder.^3^ Myolipomas are frequently found as incidental findings with CT scans and are more common in patients with thalassaemia, although not commonly in the adrenal glands.^4,11,14,17,18^

Although rare, thalassaemia does pose a risk for the development of adrenal EMH tumours, because haematopoiesis in the bone marrow is impaired and haematopoietic stem cells migrate to other tissues; clinicians should therefore remain cognisant when faced with patients suffering with haemoglobinopathies. Early detection and subsequent biopsy can be performed, where the expertise is available, to rule out serious pathology, whereby surgery can be avoided and optimum patient outcomes achieved. Incidentalomas may seem trivial and only found when small. However, EMH tumours as large as 19×15×25cm (3.5kg capsulated tumour) have been reported as palpable masses.^2,14^ Initially this tumour was detected incidentally (7.5cm), treated conservatively for 2 years and only excised surgically after the tumour grew causing symptoms.^2,8,12,14^ As reported,^2,10^ surgical treatment should be limited to patients who are symptomatic and require definitive treatment.

Keikhaei *et al*^4^ reported a case of a 26-year-old man with β-thalassaemia major, who had a non-symptomatic, non-palpable right abdominal mass. Percutaneous biopsy was performed and was suggestive of EMH. The patient refused the recommended surgery and was treated conservatively with improved transfusions and hydroxyurea, a gamma-inducer drug that supresses ineffective haematopoiesis. Hydroxyurea increases HbF synthesis, which improves the effectiveness of erythropoiesis, which in turn may help inactivate and shrink EMH tumours in patients with β-thalassaemia. EMH is reported^4^ to be most commonly seen in un-transfused thalassaemic intermedia patients and less commonly when erythropoiesis is inadequately supressed.

Comparing the case presented here with the 14 cases reported in the literature (thalassaemia), Hb levels were within normal thalassaemic range except for three patients with Hb levels of 5 to 7 mg/dl (Table 1). When considering that a fluctuating Hb level might be a factor in adrenal EMH induction, only our case showed Hb levels over a period of 3 years that were all within the normal range (Table 1). Therefore, there is insufficient evidence to exclude the possibility of a direct link between Hb and adrenal EMH. Regarding bilirubin and other laboratory measures, the patient herein showed fluctuating levels over 3 years. Again, there is insufficient evidence to link these fluctuations with EMH tumour formation (Table 1). Furthermore, our case was not an incidental asymptomatic finding because unexplained right upper quadrant pain led to further investigations, as in 7 of the 14 cases reported of thalassaemia in the literature, and one of the six patients with other haemoglobinopathies. Of the thalassaemia cases identified, only 40% presented with abdominal pain, 47% as an incidentaloma and 20% with tender/non-tender palpable mass; there were two cases with weight loss and anorexia.

## Conclusion

This paper has included all the identified cases described in the world from Qatar to Italy with only 14 (thalassaemia) and 6 (other haemoglobinopathies) reported to 2019. In many instances, adrenal EMH tumours found in patients with thalassaemia have been reported incidentally. This stresses the importance of clinicians being cognisant of the potential for adrenal EMH tumours, so that in the right circumstances adrenal EMH can be taken into account when it comes to decision-making regarding surgery. The incidental findings suggest that there may be a larger number of similar patients who have an undetected adrenal EMH mass. We believe that clinicians treating patients with thalassaemia and other haemoglobinopathies should keep in mind the potential for EMH tumour growth in the adrenal glands.
